# Establishing ground truth of polyp size, morphology, and volume using three-dimensional scanning

**DOI:** 10.1055/a-2248-1385

**Published:** 2024-03-28

**Authors:** Roupen Djinbachian, Mahsa Taghiakbari, Firas Mubaid, Chakib Yahia Rekkabi, Bibi Nuzha Noorah, Daniel von Renteln

**Affiliations:** 1 25443 Gastroenterology, Centre Hospitalier de l'Université de Montréal, Montreal, Canada; 2 177460 Gastroenterology, Centre de Recherche du Centre Hospitalier de l'Universite de Montreal, Montreal, Canada; 3 25443 Division of Internal Medicine, Centre Hospitalier de l'Université de Montréal, Montreal, Canada

Establishing ground truth of polyp size, morphology, and volume using three-dimensional scanning Comprehensive artificial intelligence (AI) solutions that should cover detecting polyp morphology, size, and volume are being developed. To train such AI solutions, it is essential to obtain high quality reference (ground truth data) on polyp morphology, size, and volume.

We explored the feasibility of three-dimensional (3D) scanning of colorectal polyps to obtain computer-aided design (CAD) 3D rendering information on polyp morphology, size, and volume. During the colonoscopy, a novel virtual scale endoscope (VSE; Scale eye, EW10-VM01; Fujifilm, Tokyo, Japan) with an integrated virtual scale function was used, which allows for polyp size measurement 11 22 33 ( Video 1Video 1 ). Two detected polyps had their size measured during the colonoscopy using VSE ( Fig. 1Fig. 1 **a,d** ), then were removed from the colon as intact en bloc specimens with a healthy resection margin. The polyps were measured using a digital Vernier caliper (eSync with 32 feeler gauge with 0.01-mm intervals) directly after resection ( Fig. 1Fig. 1 **b,e** ). The polyps were then 3D scanned for volumetric information (Artec Space Spider; Artec 3D, Luxembourg) 44 ( Fig. 1Fig. 1 **c,f** ). The 3D scanner processes up to 1 million individual points per second at 7.5 frames per second with a resolution of 0.1 mm. Polyp 1 was measured as being 8 mm using the VSE, 8.88 mm using the caliper, and 9.68 mm in the 3D model. The total polyp volume was 291.8 mm ^3^ . Polyp 2 was measured as being 14 mm using the VSE, 15.16 mm using the caliper, and 15.20 mm in the 3D model. The total polyp volume was 968.1 mm ^3^ .

Two polyps are measured using a virtual scale endoscope (VSE) and then by subsequent 3D rendering after their resection.Video 1Video 1

**Fig. 1:**
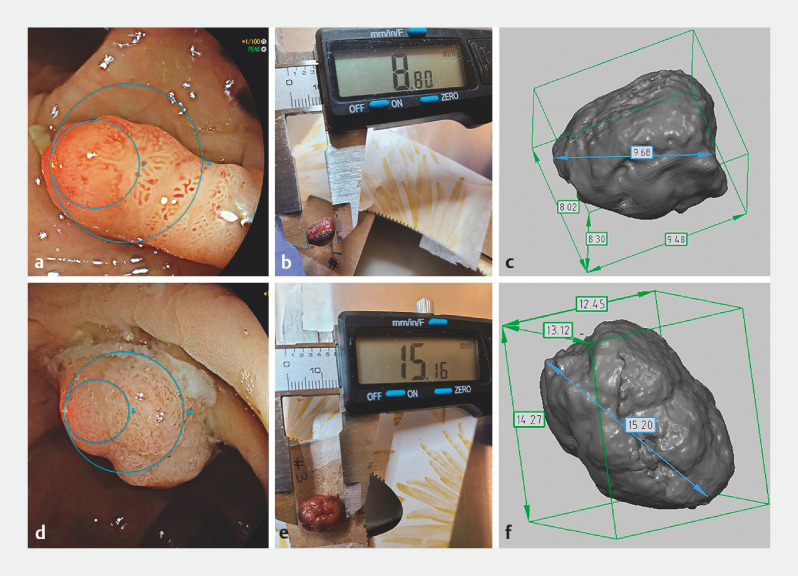
**Fig. 1** Images showing the measurement of two polyps using: **a,d** the virtual scale endoscope; **b,e** a vernier caliper; **c,f** 3D rendering after the polyps’ resection.

We found that it is possible to obtain spatial polyp information through 3D scanning. 3D scanning of polyps can capture their shape, geometries, and textures, translating them into data files in which size, volume, and shape becomes measurable. These 3D models can then be used in the context of AI development to provide ground truth data to train models to automatically recognize size, volume, and Paris classification.

Endoscopy_UCTN_Code_CPL_1AJ_2AB

Zitierweise für diesen Artikel

Endoscopy 2023; 55: E1260–E1261. doi: 10.1055/a-2210-0635

## Notes

Endoscopy E-Videos https://eref.thieme.de/e-videos
*E-Videos* is an open access online section of the journal *Endoscopy* , reporting on interesting cases and new techniques in gastroenterological endoscopy. All papers include a high-quality video and are published with a Creative Commons CC-BY license. Endoscopy E-Videos qualify for HINARI discounts and waivers and eligibility is automatically checked during the submission process. We grant 100% waivers to articles whose corresponding authors are based in Group A countries and 50% waivers to those who are based in Group B countries as classified by Research4Life (see: https://www.research4life.org/access/eligibility/ ). This section has its own submission website at https://mc.manuscriptcentral.com/e-videos .
